# Relationship between WBRT total dose, intracranial tumor control, and overall survival in NSCLC patients with brain metastases - a single-center retrospective analysis

**DOI:** 10.1186/s12885-019-6307-8

**Published:** 2019-11-14

**Authors:** Zhensheng Li, Dongxing Shen, Jian Zhang, Jun Zhang, Fang Yang, Deyou Kong, Jie Kong, Andu Zhang

**Affiliations:** Department of Radiation Oncology, the Fourth Hospital of Hebei Medical University, 169 Tianshan Street, Shijiazhuang, 050035 China

**Keywords:** Whole brain radiotherapy, Non-small cell lung cancer, Brain metastases, Overall survival, Intracranial progression-free survival

## Abstract

**Background:**

The relationship between whole brain radiotherapy (WBRT) dose with intracranial tumor control and overall survival (OS) in patients with non-small cell lung cancer (NSCLC) brain metastases (BM) is largely unknown.

**Methods:**

We retrospectively analyzed 595 NSCLC BM patients treated consecutively at the Fourth Hospital of Hebei Medical University between 2013 to 2015. We assigned the patients into 4 dose groups of WBRT: none, < 30, 30–39, and ≥ 40 Gy and assessed their relationship with OS and intracranial progression-free survival (iPFS). Cox models were utilized. Covariates included sex, age, KPS, BM lesions, extracranial metastasis, BM and lung tumor resection, chemotherapy, targeted therapy, and focal radiotherapy modalities.

**Results:**

Patients had a mean age of 59 years and were 44% female. Their median survival time (MST) of OS and iPFS were 9.3 and 8.9 months. Patients receiving none (344/58%), < 30 (30/5%), 30–39 (93/16%), and ≥ 40 (128/22%) Gy of WBRT had MST of OS (iPFS) of 7.3 (6.8), 6.0 (5.4), 10.3 (11.9) and 11.9 (11.9) months, respectively. Compared to none, other WBRT groups had adjusted HRs for OS - 1.23 (*p > 0.20*), 0.72 (*0.08*), 0.61 (*< 0.00*) and iPFS - 1.63 (*0.03*), 0.71 (*0.06*), 0.67 *(< 0.01*). Compared to 30–39 Gy, WBRT dose ≥40 Gy was not associated with improved OS and iPFS (all *p > 0.40*). Stratified analyses by 1–3 and ≥ 4 BM lesions and adjustment analyses by each prognostic index of RPA class, Lung-GPA and Lung-molGPA supported these relationships as well.

**Conclusions:**

Compared to none, WBRT doses ≥30 Gy are invariably associated with improved intracranial tumor control and survival in NSCLC BM patients.

## Background

Brain metastasis (BM) is a common complication in non-small cell lung cancer (NSCLC), affecting up to 50% of patients within the overall disease course [1, 2]. Even with the best supportive care, BM patients usually have a median survival time (MST) of only 1–2 months [3]. The BM population is extremely heterogeneous with varied outcomes signifcantly associated with the recursive partitioning analysis (RPA) classes I - III and graded prognostic assessment (GPA) criteria scores [4–6]. For decades, whole brain radiotherapy (WBRT) to control neurologic symptoms and intracranial tumor growth has been the standard treatment for NSCLC BM patients [7, 8]. In some studies, WBRT has been shown to extend patient MST up to 7 months with a range of 3 to 15 months [5, 8]. However, the relationship between total or biological effective dose (BED) of WBRT with intracranial tumor control and overall survival (OS) has not been elucidated well [9].

Supported mostly by symptom control trials, current NCCN guidelines (version.2.2018) recommend WBRT dose schemes of 20 - 40Gy/5–20 fractions (f) and 20Gy/5f for poor responders [10–13]. For patients who are oligometastatic (having 1–3 lesions) or have good GPA scores, WBRT is combined with surgical resection or stereotactic radiotherapy (SRT) to further reduce intracranial relapse and mortality [14–17]. For patients with multiple metastases (having ≥4 lesions), WBRT is preferably used; however, intensity of WBRT has severe side effects of dose-related memory decline and neurocognitive dysfunction over time should be considered when considering treatment dosage [18–21]. To resolve this dilemma, radiosensitizing or chemotherapeutic agents and hippocampal avoidance techniques have been studied in pursuit of the optimal low but still effective WBRT dose [22–25]. In this regard, the determination of minimal WBRT dose for tumor control or survival improvement is highly relevant.

This study assesses the association of WBRT total dose levels with OS and intracranial progression-free survival (iPFS) through retrospective analysis of a recent cohort of NSCLC BM patients treated at one center in China.

## Methods

### Study population

Five hundred ninety-five NSCLC BM patients who were newly and consecutively treated at the Fourth Hospital of Hebei Medical University between 2013 to 2015 were retrospectively considered and analyzed in our study. All patients received a pathological diagnosis of NSCLC based on the primary tumor and their BM diagnoses were established by CT or MRI brain imaging. Meningeal metastasis was additionally diagnosed by having imaging features of enhanced nodules or lumps of BM images or malignant cells identified in the cerebrospinal fluid. Patient were followed up every 2 to 3 months after discharge and encouraged to visit the hospital clinic immediately upon new or worsening signs or symptoms. Patients alive on December 1, 2016 were censored. Treatment failures included death or intracranial tumor progression defined as a new enhancing lesions or > 20% increase in one-dimensional measurements of an existing lesion per the Response Evaluation Criteria in Solid Tumor (RECIST) guidelines (version 1.1). The OS (iPFS) days was defined as 1 plus number of days between BM diagnosis and death date (the earlier date of treatment failures) or December 1, 2016, whichever was earliest. iPFS was considered as a proximate measure of intracranial tumor control.

### WBRT and other radiotherapy on BM

Only 42% (*n* = 251) of our study population received WBRT regardless of other RT modalities administered. Causes could be admissions and management in different clinical departments of our hospital independent of consultation with the Department of Radiation Oncology as well as some physicians lacking standardized guidelines to treat NSCLC BM.

In consideration of variable independence required in statistical models, RT modalities were classified into (1) four total dose levels of WBRT: 0 (i.e. none), < 30, 30–39, and ≥ 40 Gy; (2) three local RT dose levels of 0, < 50, and ≥ 50 Gys delivered focally or through boost RT with simultaneous or sequential WBRT to the largest BM lesion; (3) whether SRT was used or not.

All RTs were delivered with 3-dimensional conformal or intensity-modulated ones (IMRT) and used 6 MV X-rays generated by medical accelerators. Only daily RT was performed. Among WBRT patients, the delivered regimens of 40Gy/20f, 30Gy/10f, and 37.5Gy/15f constituted 46, 41, and 5% (*n* = 12), respectively; less than 3% (*n* = 6) selectively used 20Gy/5f due to initial poor performance; less than 6% failed to complete the prescribed WBRT sessions due to debilitating performance, serious adverse events (SAEs), or voluntary withdrawal. Further analysis of charted SAE events showed that over 95% SAEs were hematologic in nature (leukopenia, neutropenia, or thrombocytopenia) with Radiation Therapy Oncology Group (RTOG) toxicity grades ≥3, likely due to recent chemotherapy or chest RT. All SRTs were carried out with Gamma Knife with marginal doses of 10–15 Gy (defined to represent the 50% prescription isodose line) to the solitary or larger tumor of BM patients with 1–2 brain lesions.

### Statistical methods

Statistics were described in terms of mean, median, percentage of total, standard deviation (std), and others. Comparisons were conducted by ANOVA, Wilcoxon rank-sum, Chi-squared test or Fisher’s Exact test if applicable. Kaplan-Meier curves were used to estimate MSTs and 95% confidence intervals (CI). Proportional hazard Cox models were used to estimate hazard ratios (HR) and 95% CIs with *p* values. Final covariates were determined after examining univariate analysis results and through review of current literature. Two-sided *p* < 0.05 was cited as being statistically significant. All statistical analyses were performed with SAS 9.20.

Ethics and Informed consent.

The study was approved by the Medical Ethics Committee of the Fourth Hospital of Hebei Medical University in China in 2016 (record #: 2016–0634). No written or verbal consent from participants was needed for retrospective analyses under the Chinese Government’s medical research regulation and restrictions. Only de-identified protected health information was used.

## Results

### Comparison of patient’s characteristics among WBRT subgroups

Overall, patients had a mean age (std, range) of 58.7 (10.0, 27–82) years. 43.5% were female; 42% (*n* = 251) had WBRT. Patients were stratified into four dose levels of WBRT as mentioned previously, 0 (i.e. none), < 30, 30–39, and ≥ 40 Gy, with compositions (number) of 58% (344), 5% (30), 16% (93) and 22% (128), respectively (Table 1). In short, patients who had WBRT were more likely to have poor performance (Karnofsky Performance Score, KPS < 70), short NSCLC history (< 1 month), no extracranial metastases, radical resection of primary lung tumor, and were less likely to have de novo cTNM Stage IV (60% vs. 80%) at NSCLC diagnosis.

**Table 1:** Patient characteristics and their comparison among subgroups by WBRT dose

Variables	WBRT Dose					All
	None	< 30Gy	30-39Gy	≥40Gy		
	(n_1_ = 344)	(n_2_ = 30)	(n_3_ = 93)	(n_4_ = 128)		(*N* = 595)
	n(%)	n(%)	n(%)	n(%)	*p*^a^	N(%)
Sex						
female	159 (*46.2*)	14 (*46.7*)	30 (*32.3*)	56 (*43.8*)	*0.114*	259 (*43.5*)
male	185 (*53.8*)	16 (*53.3*)	63 (*67.7*)	56 (*56.2*)		336(*56.5*)
CVD	146 (*42.4*)	18 (*60.0*)	40 (*43.0*)	53 (*41.4*)	*ns*	257 (*43.2*)
Age (years)						
<50	61 (*17.7*)	3 (*10.0*)	13 (*14.0*)	25 (*19.5*)	*0.115*	102 (*17.1*)
50–59	104 (*30.2*)	8 (*26.7*)	36 (*38.7*)	52 (*40.6*)		200 (*33.6*)
≥ 60	179 (*52.6*)	19 (*63.3*)	44 (*47.3*)	51 (*39.8*)		293 (*49.2*)
KPS						
< 70	86 (*25.0*)	13 (*43.3*)	37 (*39.8*)	54 (*42.2*)	*< 0.001*	190 (*31.9*)
70–80	77 (*22.4*)	7 (*23.3*)	35 (*37.6*)	45 (*35.2*)		164 (*27.6*)
≥ 90	181 (*52.6*)	10 (*33.3*)	21 (*22.6*)	29 (*22.7*)		241 (*40.5*)
NSCLC history (month)						
< 1	197 (*57.3*)	12 (*40.0*)	41 (*44.1*)	51 (*39.8*)	*0.004*	301 (*50.6*)
1–6	45 (*13.1*)	5 (*16.7*)	6 (*6.5*)	22 (*17.2*)		78 (*13.1*)
6–12	45 (*13.1*)	5 (*16.7*)	17 (*18.3*)	21 (*16.4*)		88 (*14.8*)
> 12	57 (*16.6*)	8 (*26.7*)	29 (*31.2*)	34 (*26.6*)		128 (*21.5*)
BM lesion number						
1	166 (*48.3*)	11 (*36.7*)	25 (*26.9*)	36 (*28.1*)		238 (*40.0*)
2–3	53 (*15.4*)	7 (*23.3*)	18 (*19.4*)	22 (*17.2*)		100 (*16.8*)
≥ 4	125 (*36.3*)	12 (*40.0*)	50 (*53.8*)	70 (*54.7*)		257 (*43.2*)
Extracranial met.	268 (*77.9*)	19 (*63.3*)	64 (*68.8*)	84 (*65.6*)	*0.019*	435 (*73.1*)
Brain stem met.	91 (*2.4*)	1 (*7.0*)	3 (*3.2*)	3 (*2.3*)	*ns*	16 (*2.7*)
Meningeal met.	25 (*7.3*)	2 (*6.7*)	2 (*2.2*)	9 (*7.0*)	*ns*	43 (*6.4*)
Targeted therapy	97 (*28.2*)	9 (*30.0*)	23 (*24.7*)	39 (*30.5*)	*ns*	168 (*28.2*)
Chemotherapy	173 (*50.3*)	11 (*36.7*)	43 (*46.2*)	72 (*56.3*)	*0.199*	299 (*50.3*)
SRT	14 (*5.1*)	0 (*0.0*)	0 (*0.0*)	1 (*0.8*)	*0.060*	15 (*2.5*)
Local/boost RT (Gy)						
none	325 (*94.5*)	23 (*76.7*)	15 (*16.1*)	55 (*43.0*)	*< 0.001*	418 (*73.0*)
<50	8 (*2.3*)	6 (*20.0*)	3 (*3.2*)	6 (*4.7*)		23 (*3.9*)
50–59	9 (*2.6*)	1 (*3.3*)	38 (*40.9*)	42 (*32.8*)		90 (*15.1*)
≥ 60	2 (*0.6*)	0 (*0.0*)	37 (*39.8*)	25 (*19.5*)		64 (*10.8*)
BM resection						
none	326 (*94.8*)	29 (*96.7*)	90 (*96.8*)	118 (*92.2*)	*ns*	563 (*94.6*)
incomplete	2 (*0.7*)	0 (*0.0*)	0 (*0.0*)	2 (*1.5*)		4 (*0.7*)
complete	16 (*4.7*)	1 (*3.3*)	3 (*3.2*)	8 (*6.3*)		28 (*4.7*)
Initial cTNM Stage IV	274 (*79.7*)	21 (*70.0*)	55 (*59.1*)	75 (*58.6*)	*< 0.001*	425 (*71.4*)
Lung tumor surgery						
none	301 (*87.5*)	23 (*76.7*)	67 (*72.0*)	90 (*70.3*)	*< 0.001*	481 (*80.8*)
incomplete	6 (*1.7*)	3 (*10.0*)	4 (*4.3*)	11 (*8.6*)		24 (*4.0*)
radical	37 (*10.8*)	4 (*13.3*)	22 (*23.7*)	27 (*21.1*)		90 (*15.1*)
Adenocarcinoma	242 (7*0.3*)	22 (*73.3*)	66 (*71.0*)	91 (*71.1*)	*ns*	421 (*70.8*)
EGFR mutation						
neg.	55 (*16.0*)	3 (*10.0*)	23 (*24.7*)	20 (*15.6*)	*ns*	101 (*17.0*)
pos.	74 (*21.5*)	5 (*16.7*)	13 (*14.0*)	22 (*17.2*)		114 (*19.2*)
no record	215 (*62.5*)	22 (*73.3*)	57 (*61.3*)	86 (*67.2*)		380 (*63.9*)
ALK mutation						
neg.	15 (*4.4*)	1 (*3.3*)	3 (*3.2*)	3 (*2.3*)	*0.069*	22 (*3.7*)
pos.	1 (*0.3*)	2 (*6.7*)	2 (*2.2*)	1 (*0.8*)		6 (*1.0*)
no record	328 (*95.3*)	27 (*90.0*)	88 (*94.6*)	124 (*96.9*)		567 (*95.3*)

*WBRT* whole brain radiotherapy; *CVD* cardiovascular disease; *ns* not significant with *p* > *0.20*; *BM* brain metastasis; *KPS* Karnofsky Performance Score; *NSCLC* non-small cell lung cancer; *met.* Metastases; *SRT* stereotactic radiotherapy; *RT* radiotherapy; *EGFR* epidermal growth factor receptor; *neg.* Negative; *pos.* Positive; *ALK* anaplastic lymphoma kinase.

^a^ from the Chi-square or Fisher’s exact (if applied) test.

### Survivals of overall and by WBRT subgroups

**Fig. 1:**
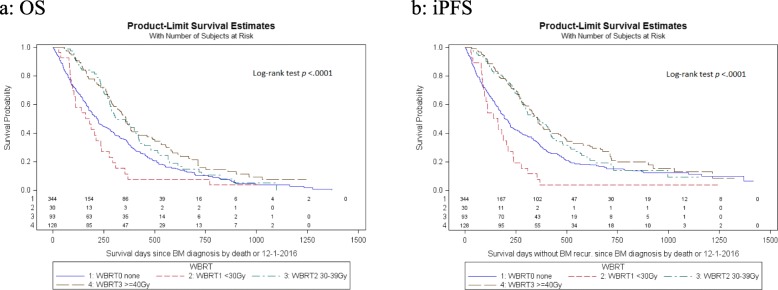
Kaplan-Meier overall (OS) and intracranial progression-free survival (iPFS) curves respectively (**a** & **b**) for the entire cohort of study population by the whole-brain radiation therapy (WBRT) use status. Risk mentioned in the figure titles means the WBRT status

Overall, the estimated MST (95% CI) of OS and iPFS were 9.3 (8.3–10.0) and 8.9 (7.6–9.6) months. Figure 1 shows the Kaplan-Meier curves of OS and iPFS of the four WBRT dose groups (both log-rank test *p* < *0.001*) 0, < 30, 30–39, and ≥ 40 Gy. The MST of OS (iPFS) were 7.3 (6.8), 6.0 (5.4), 10.3(11.9) and 11.9 (11.9) months, respectively. Pair-wise comparisons showed non-WBRT patients had worse OS and iPFS than WBRT patients with doses of 30–39 Gy or ≥ 40 Gy (both *p* < *0.001*). No statistical differences of OS and iPFS between patients with WBRT 30–39 Gy and ones with WBRT ≥40 Gy (both *p > 0.50*) were found. Estimated one-year survival rates were 37, 11, 62, and 63% for OS and 29, 11, 62, and 62% for iPFS, respectively.

### Univariate and multivariable survival analyses

Table 2 shows the univariate analysis results. Except for SRT, cardiovascular disease (CVD), BM lesion number, BM resection, initial cTNM Stage IV, many characteristics were found to be statistically associated with OS or iPFS (*p < 0.05*). Compared to no treatment, WBRT 30–39 Gy and ≥ 40Gy were found to be significantly associated with improved OS or iPFS.

**Table 2:** Univariate Cox model analyses

Variables	OS			iPFS			
	HR	95%CI	*p*^a^	HR	95%CI		*p*^a^
WBRT (Gy)							
none	1.000		*ref.*	1.000			*ref.*
< 30	1.418	(0.938–2.142)	*0.098*	1.713	(1.133–2.588)		*0.011*
30–39	0.709	(0.540–0.933)	*0.014*	0.694	(0.533–0.904)		*0.007*
≥ 40	0.616	(0.483–0.785)	*< 0.001*	0.640	(0.506–0.809)		*< 0.001*
Local/boost RT (Gy)							
none	1.000		*ref.*	1.000			*ref.*
< 50	0.722	(0.422–1.234)	*ns*	0.977	(0.608–1.572)		*ns*
50–59	0.575	(0.436–0.759)	*< 0.001*	0.668	(0.515–0.866)		*0.002*
≥ 60	0.594	(0.427–0.825)	*0.002*	0.566	(0.409–0.783)		*< 0.001*
SRT	0.713	(0.365–1.391)	*ns*	0.645	(0.344–1.208)		*ns*
Female	0.761	(0.627–0.924)	*0.006*	0.754	(0.626–0.909)		*0.003*
CVD	0.970	(0.801–1.175)	*ns*	1.039	(0.863–1.250)		*ns*
Age (years)							
< 50	1.000		*ref.*	1.000			*ref.*
50–59	1.051	(0.783–1.412)	*ns*	0.889	(0.673–1.174)		*ns*
≥ 60	1.363	(1.038–1.791)	*0.052*	1.169	(0.904–1.512)		*ns*
KPS							
< 70	1.000		*ref.*	1.000			*ref.*
70–80	0.920	(0.718–1.178)	*ns*	0.918	(0.722–1.166)		*ns*
≥ 90	0.765	(0.610–0.958)	*0.020*	0.842	(0.676–1.047)		*ns*
NSCLC history (month)							
< 1	0.910	(0.710–1.167)	*ns*	1.023	(0.805–1.299)		*ns*
1–6	1.153	(0.834–1.595)	*ns*	1.410	(1.030–1.931)		*0.032*
6–12	1.107	(0.799–1.533)	*ns*	1.236	(0.972–1.693)		*0.187*
> 12	1.000		*ref.*	1.000			*ref.*
BM lesion number							
1	1.000		*ref.*	1.000			*ref.*
2–3	1.103	(0.833–1.461)	*ns*	1.053	(0.804–1.379)		*ns*
≥ 4	1.118	(0.907–1.377)	*ns*	1.099	(0.898–1.344)		*ns*
Extracranial met.	1.244	(0.988–1.565)	*0.063*	1.695	(1.356–2.120)		*< 0.001*
Brain stem met.	2.185	(1.226–3.896)	*0.008*	1.901	(1.069–3.381)		*0.029*
Meningeal met.	1.212	(0.853–1.723)	*ns*	1.501	(1.063–2.119)		*0.021*
Targeted therapy	0.492	(0.393–0.617)	*< 0.001*	0.597	(0.484–0.736)		*< 0.001*
Chemotherapy	0.612	(0.506–0.741)	*< 0.001*	0.791	(0.659–0.951)		*0.013*
BM resection							
none	1.000		*ref.*	1.000			*ref.*
incomplete	0.786	(0.196–3.157)	*ns*	0.808	(0.201–3.245)		*ns*
complete	0.882	(0.556–1.398)	*ns*	0.851	(0.549–1.320)		*ns*
Initial cTNM Stage IV	1.027	(0.829–1.271)	*ns*	1.106	(0.901–1.357)		*ns*
Lung tumor surgery							
none	1.000		*ref.*	1.000			*ref.*
incomplete	0.842	(0.524–1.353)	*ns*	0.865	(0.546–1.372)		*ns*
radical	0.917	(0.687–1.225)	*ns*	0.695	(0.527–0.916)		*0.001*
Adenocarcinoma	0.809	(0.659–0.993)	*0.043*	0.821	(0.672–1.002)		*0.053*
EGFR mutation							
neg.	1.000		*ref.*	1.000			*ref.*
pos.	0.670	(0.484–0.927)	*0.016*	0.625	(0.460–0.850)		*0.003*
no record	1.195	(0.931–1.534)	*0.161*	0.962	(0.756–1.226)		*ns*
ALK mutation							
neg.	1.000		*ref.*		1.000		*ref.*
pos.	0.415	(0.118–1.458)	*0.170*		1.059	(0.351–3.191)	*ns*
no record	1.006	(0.578–1.750)	*ns*		1.109	(0.663–1.857)	*ns*

*OS* overall survival; *iPFS* intracranial progression-free survival; *HR* hazard ratio; *95%CI* 95% confidence interval; *WBRT* whole brain radiotherapy; *ref.* reference; *RT* radiotherapy; *ns* not significant with *p > 0.20*; *SRT* stereotactic radiotherapy; *CVD* cardiovascular disease; *BM* brain metastasis; *KPS* Karnofsky Performance Score; *NSCLC* non-small cell lung cancer; *met. M*etastases; *EGFR* epidermal growth factor receptor; *neg.* Negative; *pos.* Positive; *ALK* anaplastic lymphoma kinase.

^a^ from the univariate Cox model analysis.

Table 3 shows the multivariable analysis results. Tumor pathology (adenocarcinoma vs. non-adenocarcinoma) was not included as a covariate because it was insignificantly associated with OS and iPFS, with adjusted HRs of 1.066 (*p = 0.59*) and 0.965 (*p* = *0.75*), respectively. Compared to none, both WBRT 30–39 Gy and ≥ 40 Gy were associated with improved OS, with HRs of 0.722 (*p = 0.08*) and 0.609 (*p* < *0.01*) respectively, and with improved iPFS, with HRs of 0.714 (*p = 0.06*) and 0.669 (*p* < *0.01*) respectively. However, patients with WBRT ≥40 Gy and 30–39 Gy showed no significant difference of OS (HR 0.843, *p = 0.34*) and iPFS (HR 0.937, *p = 0.70*). If the dose-effect of WBRT is assumed to be in one direction and continuous, WBRT doses ≥30 Gy appear to be invariably associated with improved intracranial tumor control and survival in NSCLC BM patients. Compared to none, local RT dose ≥50 Gy and SRT were significantly associated with improved OS. Compared to none, the local RT dose ≥60 Gy was significantly associated with improved iPFS (*p = 0.03*).

**Table 3:** Multivariable Cox model analyses

Variables	OS			iPFS		
	HR	95%CI	*p*^a^	HR	95%CI	*p*^a^
WBRT (Gy)						
none	1.000		*ref.*	1.000		*ref.*
< 30	1.227	(0.787–1.914)	*ns*	1.625	(1.045–2.528)	*0.031*
30–39	0.722	(0.500–1.042)	*0.082*	0.714	(0.502–1.017)	*0.062*
≥ 40	0.609	(0.453–0.818)	*0.001*	0.669	(0.500–0.895)	*0.007*
Local/boost RT (Gy)						
none	1.000		*ref.*	1.000		*ref.*
< 50	0.609	(0.341–1.088)	*0.042*	0.930	(0.555–1.561)	*ns*
50–59	0.572	(0.402–0.815)	*0.003*	0.776	(0.551–1.092)	*0.146*
≥ 60	0.580	(0.385–0.873)	*0.019*	0.641	(0.427–0.963)	*0.032*
SRT	0.653	(0.326–1.308)	*0.022*	0.708	(0.364–1.376)	*ns*
Female	0.798	(0.644–0.988)	*0.039*	0.804	0.655–0.987)	*0.037*
CVD	0.868	(0.706–1.068)	*0.182*	0.936	(0.765–1.145)	*ns*
Age (years)						
< 50	1.000		*ref.*	1.000		*ref.*
50–59	1.089	(0.799–1.486)	*ns*	0.854	0.638–1.142)	*ns*
≥ 60	1.335	(0.990–1.800)	*0.058*	1.088	0.820–1.444)	*ns*
KPS						
< 70	1.000		*ref.*	1.000		*ref.*
70–80	0.987	(0.762–1.279)	*ns*	0.964	(0.748–1.241)	*0.774*
≥ 90	0.620	(0.479–0.801)	*0.000*	0.670	(0.521–0.862)	*0.002*
NSCLC history (month)						
< 1	0.632	(0.473–0.844)	*0.002*	0.780	(0.595–1.023)	*0.072*
1–6	0.895	(0.626–1.280)	*ns*	1.249	(0.891–1.750)	*0.197*
6–12	0.741	(0.525–1.045)	*0.087*	0.828	(0.590–1.160)	*ns*
> 12	1.000		*ref.*	1.000		*ref.*
BM lesion number						
1	1.000		*ref.*	1.000		*ref.*
2–3	1.181	(0.870–1.602)	*ns*	1.023	(0.766–1.366)	*ns*
≥ 4	1.325	(1.045–1.681)	*0.020*	1.148	(0.911–1.447)	*ns*
Extracranial met.	1.313	(1.019–1.691)	*0.035*	1.836	(1.428–2.361)	*0.000*
Brain stem met.	1.219	(0.664–2.235)	*ns*	1.009	(0.548–1.857)	*ns*
Meningeal met.	0.935	(0.633–1.382)	*ns*	1.136	(0.772–1.670)	*ns*
Targeted therapy	0.373	(0.290–0.480)	*0.000*	0.506	(0.402–0.636)	*0.000*
Chemotherapy	0.587	(0.476–.724)	*0.000*	0.724	(0.592–0.885)	*0.002*
BM resection						
none	1.000		*ref.*	1.000		*ref.*
incomplete	0.834	(0.200–3.482)	*ns*	0.854	(0.206–3.532)	*ns*
complete	0.709	(0.429–1.171)	*0.180*	0.909	0.557–1.486)	*ns*
Lung tumor surgery						
none	1.000		*ref.*	1.000		*ref.*
incomplete	0.889	(0.533–1.482)	*ns*	0.911	(0.559–1.483)	*ns*
radical	0.773	(0.553–1.080)	*0.132*	0.705	(0.508–0.978)	*0.036*

*OS* overall survival; *iPFS* intracranial progression-free survival; *HR* hazard ratio; *95%CI* 95% confidence interval; *WBRT* whole brain radiotherapy; *ref.* reference; *ns* not significant with *p > 0.20*; *RT* radiotherapy; *SRT* stereotactic radiotherapy; *CVD* cardiovascular disease; *BM* brain metastases; *KPS* Karnofsky Performance Score; *NSCLC* non-small cell lung cancer; *met. M*etastases.

^a^ from the multivariable Cox model analysis.

The significantly worse iPFS (HR 1.625, *p = 0.03*) associated with WBRT < 30Gy (vs. none) was an unexpected finding. Uncorrected selective bias and or confounding effects by those unadjusted or uncollected covariates could exist. In addition, patients with WBRT < 30Gy (*n* = 30) either had WBRT 20Gy/5f (*n* = 6, all KPS < 60) or withdrew prior to completing the full WBRT with planned ≥30G dose due to the most commonly worsening KPS (related or unrelated to CNS symptoms). This observed association should be regarded as reverse correlation rather than causation. To the best of our knowledge, there are no published pathological mechanisms or studies supporting the role of WBRT accelerating the dying process. The dose-effect profile of WBRT under 30 Gy merits further investigation best in randomized controlled studies.

### Stratified multivariable survival analyses by BM lesion number

To further examine if WBRT dose-effect survival profiles in oligometastatic patients could present differently from multiple metastatic ones, two sets of stratified multivariable survival analyses were conducted. Table 4 shows that WBRT 30–39 Gy and WBRT ≥40 Gy had no different effect on OS and iPFS in each subset of BM lesions (all *p* ≥ *0.50*). Compared with the non-stratified analyses, smaller stratified analysis sizes generated slightly higher *p* values (*p = 0.05–0.20*) of WBRT 30–39 Gy with improved OS and iPFS (HR = 0.59–0.78) as compared to non-WBRT patients.

**Table 4:** Stratified multivariable Cox model analyses

BM Lesions	WBRT (Gy)	OS			iPFS		
		HR	95%CI	*p*^a^	HR	95%CI	*p*^a^
1–3 (N_1_ = 338)	none	1.000		*ref.*	1.000		*ref.*
	< 30	1.607	(0.876–2.946)	*0.125*	1.660	(0.915–3.009)	*0.095*
	30–39	0.781	(0.432–1.413)	*ns*	0.700	(0.408–1.201)	*0.196*
	≥40	0.639	(0.403–1.012)	*0.056*	0.592	(0.380–0.924)	*0.021*
	≥40 vs. 30–39 (ref.)	0.818	(0.456–1.466)	*0.499*	0.846	(0.505–1.417)	*0.526*
≥4 (N_2_ = 257)	none	1.000		*ref.*	1.000		*ref.*
	< 30	0.977	(0.487–1.957)	*ns*	1.880	(0.915–3.863)	*0.086*
	30–39	0.589	(0.348–0.996)	*0.048*	0.699	(0.421–1.159)	*0.165*
	≥40	0.514	(0.328–0.806)	*0.004*	0.624	(0.401–0.969)	*0.036*
	≥40 vs. 30–39 (ref.)	0.873	(0.540–1.413)	*0.581*	0.892	(0.558–1.426)	*0.634*

*BM* brain metastases; *WBRT* whole brain radiotherapy; *OS* overall survival; *iPFS* intracranial progression-free survival; *HR* hazard ratio; *95%CI* 95% confidence interval; *ns* not significant with *p >0.20*; *ref.* reference

^a^ from the multivariable Cox model analysis without BM lesion group as one covariate

### Adjustment survival analyses by common prognostic index

The RPA, Lung-GPA, and Lung-molGPA scores are user-friendly prognostic indice in NSCLC BM patients. Their calculation formula are reported in literature [6, 20]. Table 5 shows all three prognostic indices predicted OS well for our Chinese cohort. In addition, each adjustment model by prognostic index and RT modalities shows that WBRT 30–39 Gy and ≥ 40 Gy provided no statistically different HRs of OS (all *p > 0.25*). The same conclusion was reached for HRs of iPFS (data not shown). Thus, use of these prognostic indices as one integrated covariate supported the conclusions above as well.

**Table 5:** Prognostic index adjusted Cox models

Prognostic Index			OS		Univariate		Prognostic Index & RT modality^a^ adjusted				
	n	(%)	MTS	*p*^b^	HR	*p*^c^	HR	*p*^c^	WBRT (Gy)	HR	*p*^c^
RPA class											
I	18	(3)	14.1	*0.039*	0.435	*0.032*	0.545	*0.122*	none	1.000	*ref.*
II	387	(65)	9.5		0.839	*0.094*	0.703	*0.001*	< 30	1.412	*0.120*
III	190	(32)	8.7		1.000	*ref.*	1.000	*ref.*	30–39	0.969	*0.861*
									≥40	0.725	*0.019*
									≥40 vs. 30–39	0.820	*0.270*
Lung-GPA score											
0–1.0	217	(36)	8.3	*0.001*	1.000	*ref.*	1.000	*ref.*	none	1.000	*ref.*
1.5–2.0	244	(41)	8.5		0.935	*0.528*	0.910	*0.387*	< 30	1.497	*0.072*
2.5–3.0	117	(20)	13.0		0.615	*0.001*	0.580	*0.000*	30–39	0.919	*0.644*
3.5–4.0	17	(3)	17.2		0.478	*0.031*	0.511	*0.051*	≥40	0.731	*0.023*
									≥40 vs. 30–39	0.864	*0.409*
Lung-molGPA score											
0–1.0	169	(28)	7.0	*0.000*	1.000	*ref.*	1.000	*ref.*	none	1.000	*ref.*
1.5–2.0	289	(49)	8.9		0.715	*0.002*	0.629	*0.000*	< 30	1.673	*0.026*
2.5–3.0	126	(21)	12.7		0.521	*0.000*	0.453	*0.000*	30–39	0.890	*0.516*
3.5–4.0	11	(2)	25.0		0.259	*0.008*	0.186	*0.001*	≥40	0.697	*0.009*
									≥40 vs. 30–39	0.845	*0.342*

*OS* overall survival; *RT* radiotherapy; *MTS* median survival time in months; *WBRT* whole brain radiotherapy; *HR* hazard ratio; *ref.* reference; RPA the recursive partitioning analysis; *GPA* the graded prognostic assessment; *Lung-GPA* the lung cancer-specific GPA; *Lung-molGPA* the lung cancer-specific GPA using molecular markers.

^a^RT modalities included WBRT, local/boost RT and SRT.

^b^from the log-rank test.

^c^from the Cox model analysis

## Discussion

WBRT has been used as a standard treatment for BM patients for decades. However, the relationships of WBRT total dose with intracranial tumor control and survival are rarely studied in NSCLC BM patients alone. These profiles are significantly complicated by factors of age, KPS, tumor type, BM lesion number, extracranial metastatic status, local RT modalities, among others [26]. Through this retrospective multivariable analysis, we found that compared to none, WBRT dose ≥30 Gy was invariably associated with improved OS and iPFS. This finding further warrants clinical trials for confirmation. Whether and how the lower WBRT < 30 Gy provide benefits is still unknown and should be further investigated in controlled studies.

This study used a recent large dataset from the real world. Due to differences in healthcare system and socio-cultural reasons, WBRT was administered for only 43% of all NSCLC BM patients newly treated in 2013–2015 at a single cancer institution in China. In this study, patients with WBRT 30–39 and ≥ 40 Gy had estimated MTS of OS of 10.3 and 11.9 months, respectively. Compared to non-WBRT patients, patients with WBRT ≥30 Gy had extended MTS of 4.5–6 months. Similar survival results have also been reported in other Chinese studies [27, 28]. Xiang et al. reported that 135 NSCLC BM patients with WBRT-based combined therapies had MTS of OS as 9.3 months, 1-year and 2-year survival rates as 46.3 and 16.1%, respectively [27]. Zhu et al. reported that 29 inoperable NSCLC BM patients treated with WBRT 40 Gy/20f plus simultaneous in-field boost IMRT 20Gy/5f had estimated MTS of OS and of iPFS as both 10 months [28]. Neither of two studies above enrolled non-WBRT patients. In our study, there were 58% (*n* = 344) NSCLC BM patients without WBRT as the analysis control.

Whether and how WBRT improves survivals of BM patients at low dose is a difficult question. Further studies on pathophysiology and radiobiological mechanisms of WBRT on BM are required. Through the most recent Cochrane database systematic review, Tsao et al. concluded that the HR of OS with lower biological WBRT doses as compared with control of 30Gy/10f was 1.21 (1.04–1.40, *p = 0.01*) and with higher biological WBRT doses vs. 30 Gy/10f was 0.97 (0.83–1.12, *p = 0.65*); both are regarded to have “moderate-certainty” evidences [20]. In addition to WBRT dose, many other multifactorial and interrelated complexes can contribute to survival: such as genetic mutation and blood-brain barrier interactions with local treatments (e.g. RT or surgery) or drugs [20, 26, 27]. Thus far, WBRT administered after local surgery or SRT for patients with 1–3 BM has been evidenced to reduce neurologic death and intracranial relapse but not overall mortality [14, 29]. Currently, many studies have indicated a tendency of longer OS for WBRT-based RT regimens compared to chemotherapeutic ones [18, 20]. However, in the recently published QUARTZ trial, Mulvenna et al. concluded that WBRT provides no better survival than optimal supportive care (OSC) in NSCLC BM patients considered unsuitable for surgical resection or SRT [30]. In this trial, 538 patients in 2007–2014 were randomly assigned into OSC or OSC + WBRT (20Gy/5fr) arms; both arms had the similar MSTs (8.5 and 9.2 weeks, respectively) with an insignificant HR of 1.06 (95% CI 0.90–1.26, *p* = *0.81*) [30]. We noticed that the QUARTZ trial treatment regimens served more palliative than curative purposes and that BM patients were recruited over 8 years and had quite short life expectancy period. Nonetheless, we believe our study population was far more representative of the real world of NSCLC BM patients in recent years and the conclusion should be applicable to the general NSCLC BM patients.

Many trials have failed to define the optimal dose and schedule of WBRT for OS or tumor control [7, 18, 20]. Most of them used various dose-fractionation schedules of WBRT 20–40 Gy/10 - 20f and had different endpoints making comparison and generalization of the dose-effect profile difficult. Indeed, given that WBRT dose of either 30Gy or 40Gy is biologically regarded to be well below the lethal RT dose (presumably over 50 Gy) of tumor, the majority of WBRT regimens in those trials were intended only for palliative purposes [7, 11, 12, 18]. Two RTOG trials in the early 1970s each enrolling over 900 BM patients had concluded that multiple WBRT schedules (low vs. high of 20–40 Gy) and time periods (short vs. long of 2 to 4 weeks) had similar tumor response rate, palliative effects, and time to progression and survival [11]; randomly-added ultra-short WBRT schedules (10Gy/1f vs. 12Gy/2f vs. 20Gy/5f) led to the same survival time but shorter time to brain tumor progression [12]. Kurtz et al. conducted one randomized control trial (RCT) in 255 highly-selected BM patients with good prognosis to conclude that WBRT 50Gy/20f and 30Gy/10f schedules had similar effects of symptom palliation, time to progression, cause of death, and survival [31]. Another trial comparing WBRT 32Gy plus 24.4 Gy to a boost field in 1.6 Gy fractions (b.i.d.) with WBRT 30Gy/10f among 445 patients had demonstrated that the accelerated hyper-fraction of WBRT made no difference on survival time [32]. However, one trial indicated that WBRT 40Gy/20f (b.i.d) in 113 patients had similar OS but higher tumor control rate (56% vs. 36%) and lower neurological mortality (32% vs. 52%, *p = 0.03*) than WBRT 20Gy/4f, [33]. Another trial involving 533 patients showed that WBRT 30Gy/10f compared to WBRT 12Gy/2f had a slight but statistically better OS (*p = 0.04*) [10].. These trials support our conclusion that WBRT doses ≥30 Gy provide better intracranial tumor control.

How the local treatment of BM (surgery, SRT or boost RT) impacts the dose-effect survival profiles of WBRT is infrequently studied. Some published trials showed that combining SRT or surgery with fixed-dose-schedule of WBRT had improved OS and reduced local failure in patients with single metastasis only [16, 34]. Andrews et al. conducted one RCT of 333 patients with 1–3 BM lesions and found that compared to WBRT alone, SRS + WBRT (37.5Gy/15f) had a better local control rate at 1 year follow-up (82% vs. 71%, *p = 0.01*) and better OS for single metastasis patients only (MTS 6.5 vs. 4.9 months, *p = 0.04*) but not in the entire cohort (6.5 vs. 5.7 months, *p = 0.14*); for NSCLC BM patients only, their MTS of ‘SRS + WBRT’ and ‘WBRT alone’ patients were estimated as 5.0 vs. 3.9 months (*p = 0.05*), respectively [16]. Patchell et al. conducted another RCT by assigning 48 patients with single BM into surgery + WBRT (36 Gy/12f) vs. WBRT alone and found significant advantages of lower local failure (20% vs. 52%, *p < 0.02*) and longer MTS (40 weeks vs. 15 weeks, *p < 0.01*) for the surgery + WBRT patients [34]. However, one trial by Mintz et al. failed to show the benefit of improving OS (MST 5.6 months vs. 6.3 months, *p = 0.24*) by having surgery first for the single BM patients who had the universal WBRT 30Gy/10f [35]. To determine the effects of adding boost RT to WBRT, Antoni et al. retrospectively analyzed 208 BM patients (137 from lung cancer) with RPA II and 1–2 metastases and found that patients with boost RT 9Gy/3f had MST of 2.2 months longer (5.9 vs. 3.7 months, *p = 0.03*) and higher local tumor control rates at 6-, 12- and 24-month (*p = 0.03*) than patients with WBRT (30Gy/10f) alone [36]. In this study, we had 15 SRT patients (only one had subsequent WBRT) and 32 surgical patients (14 of them had WBRT before or after BM surgery). Through multivariable analyses, we found that SRT was associated with better OS but not iPFS, and the boost ≥50 Gy was associated with better OS than iPFS (Table 3).

Other factors affecting OS and iPFS were also identified in this study. Chemotherapy and targeted therapy were found to be quite effective in improving OS and iPFS (*p < 0.001*). While female, young age, good KPS, short NSCLC history, and primary tumor resection were associated with improved survival, the presence of extracranial metastasis and BM lesions ≥4 predicted poorer survival. These findings were consistent with other studies [4, 37–42]. In this study, instead of using calculated GPA or RPA score, we decided to use individual covariates in Cox models to better estimate the independent dose-survival effect of WBRT. The adjustment analyses by RPA, Lung-GPA or Lung-molGPA confirmed that OS and iPFS profiles of WBRT dose level have not changed. The survival profiles of these common prognostic indices were also found to be consistent with other studies [4, 6, 41].

We recognize that our current study has both limitations and strengths. In addition to the hidden selection biases of any retrospective analysis, weaknesses include: (1) the resultant link of delivered ‘RT boost’ and higher WBRT dose could compromise their independent benefit profile evaluation in somewhat way even through multivariate and stratified analyses; (2) the BED of WBRT was not calculated for use; we were concerned with the accuracy and validity of using traditional linear-quadratic formula and citing a specific α/β value for BED calculation among these NSCLC BM patients who received heterogeneous modalities of RT rather than the fixed-schedule of universal WBRT; as aforementioned, the actual percents of 40Gy/20f, 30Gy/10f, and 37.5Gy/15f regimen used were 46, 41, and 5% in 251 WBRT patients; (3) neither neurologic symptoms nor quality of life measurements were collected; (4) Only 4.7% of patients took the ALK gene mutation test; how this low test rate, high positive rate (21%, 6/28) and the rare use of ALK drugs in the Chinese population impact the study results was difficult to assess. Strengths of this study include (1) our cohort study was conducted at a single center between 2013 to 2015 during which the guidelines of NSCLC BM treatment experienced little variation; (2) three other RT modalities in their independent formats were considered in multivariable analyses; (3) individual covariates were also presented in the final models.

## Conclusions

We conclude that compared to none, WBRT doses ≥30 Gy are invariably associated with improved intracranial tumor control and survival in NSCLC BM patients.

## Data Availability

The de-identified analysis datasets can be available from the corresponding author once the manuscript has been accepted for publication with the approval of the Fourth Hospital of Hebei Medical University in China.

## Abbreviations

ALK: Anaplastic lymphoma kinase
BED: Biological effective dose
BM: Brain metastases
CI: Confidence interval
CVD: Cardiovascular disease
EGFR: Epidermal growth factor receptor
f: Fractions
GPA: Graded prognostic assessment
HR: Hazard Ratio
IMRT: Intensity-modulated radiotherapy
iPFS: Intracranial progression-free survival
KPS: Karnofsky Performance Score
MST: Median survival time
NSCLC: Non-small cell lung cancer
OS: Overall survival
OSC: Optimal supportive care
RCT: Randomized control trial
RECIST: Response Evaluation Criteria in Solid Tumor
RPA: Recursive partitioning analysis
RT: Radiotherapy
SAE: Serious adverse events
SRT: Stereotactic radiotherapy
std.: Standard deviation
WBRT: Whole brain radiotherapy
